# Adolescent Male with Severe Groin Pain Due to Traumatic Injury

**DOI:** 10.5811/cpcem.2020.12.49730

**Published:** 2021-03-24

**Authors:** Hirofumi Ohno, Shinsuke Takeda, So Mitsuya, Ken-ichi Yamauchi

**Affiliations:** * Toyohashi Municipal Hospital, Department of Orthopedic Surgery, Toyohashi Aichi, Japan; † Toyohashi Municipal Hospital, Trauma and Microsurgery Center, Toyohashi Aichi, Japan

**Keywords:** Traumatic iliacus hematomas, Iliacus hematomas

## Abstract

**Case Presentation:**

A 14-year-old boy presented to the emergency department complaining of severe groin pain on the right side following a minor fall. Computed tomography and magnetic resonance imaging revealed a hematoma in his right iliacus muscle. He was diagnosed with a traumatic iliacus hematoma, and he recovered spontaneously with short-term oral analgesics.

**Discussion:**

Traumatic iliacus hematomas are rare entities and subside with conservative management in most cases. However, this condition may be associated with femoral nerve palsy, and surgery is indicated in severe cases. Traumatic iliacus hematoma should be considered in the differential diagnosis of severe groin pain.

## CASE PRESENTATION

**Image 1:**
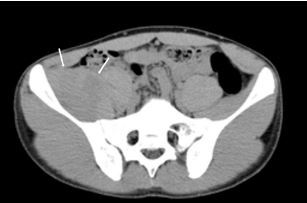
Contrast-enhanced computed tomography showing the enlarged right iliacus muscle (arrows).

**Image 2:**
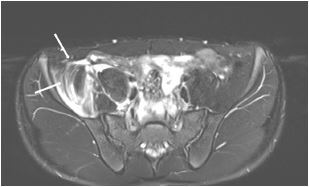
T2-weighted magnetic resonance imaging showing a high-intensity lesion in the right iliac muscle (arrows).

A 14-year-old boy with no past medical history presented to the emergency department (ED) complaining of severe groin pain on the right side following a minor fall incurred while playing handball. On physical examination, he could not actively move his right hip and was unable to walk. He had no neurological symptoms. Computed tomography (CT) showed swelling of the right iliacus muscle (Image 1). Therefore, magnetic resonance imaging (MRI) was performed to confirm this mass (Image 2). The final diagnosis was traumatic iliacus hematoma.

## DISCUSSION

Iliacus hematomas are rare, and but they are often complicated in patients with hemophilia and in those receiving anticoagulants^1^; however, some cases of post-traumatic hematomas have been reported previously.^2^,^3^ The most prevalent cause of iliacus hematomas is traumatic injuries, particularly those that are sports-related and that occur in young patients.^2^ Such injuries are often complicated by femoral nerve palsy. Because the femoral nerve travels between the psoas and iliacus muscles, hematoma of these muscles tends to compress the femoral nerve.^1^ This condition is clinically characterized by weakness of the iliopsoas muscle, loss of the knee-jerk reflex, and anteromedial thigh hypoesthesia.^4^

Conservative management is preferred for patients with mild symptoms, as the hematoma may subside by the tamponade effect. However, if symptoms progress, invasive interventions such as CT-guided drainage and surgical decompression should be considered. In this case, two weeks of oral acetaminophen alleviated the symptoms. Emergency physicians should consider traumatic iliacus hematoma in the differential diagnoses of severe groin pain, regardless of the patient’s medical history.

**CPC-EM Capsule**

What do we already know about this clinical entity?

*Traumatic iliacus hematoma is a benign and rare condition caused by traumatic injuries, especially those that are sports related. In some cases, it might be complicated by femoral nerve palsy.*

What is the major impact of the image(s)?

*Computed tomography is not enough for diagnosis of iliacus hematoma. If this condition is suspected, a magnetic resonance imaging should be performed.*

How might this improve emergency medicine practice?

*Emergency physicians should consider traumatic iliacus hematoma in the differential diagnosis of severe groin pain. Regarding our case, those who have no medical condition can suffer traumatic iliacus hematoma.*
